# Glucocorticoid replacement therapy for primary and secondary adrenal insufficiency and their impact on cognition

**DOI:** 10.3389/fendo.2023.1153595

**Published:** 2023-03-17

**Authors:** Birgit Harbeck, Peter Kropp, Ilonka Kreitschmann-Andermahr

**Affiliations:** ^1^ III. Department of Medicine, University Medical Center Hamburg-Eppendorf, Hamburg, Germany; ^2^ Medizinisches Versorgungszentrum (MVZ) Amedes Experts, Endocrinology, Hamburg, Germany; ^3^ Institute of Medical Psychology and Medical Sociology, University of Rostock, Rostock, Germany; ^4^ Department of Neurosurgery and Spine Surgery, University Hospital Essen, Essen, Germany

**Keywords:** adrenal insufficiency, glucocorticoids, replacement therapy, cognition, sleep

## Abstract

Patients with adrenal insufficiency (AI) are treated with conventional or modified-release glucocorticoid (GC) replacement therapy (GRT). Although current GRT regimens aim to mimic the physiological circadian pattern of cortisol secretion, temporary phases of hypo- and hypercortisolism are common. There is good evidence that prolonged phases of hypo- or hypercortisolism are associated with impaired cognitive functioning. However, little is known about cognitive functioning in patients with AI regarding the effects of dosage and duration of glucocorticoid replacement therapy. There is also little data available comparing the effects of GC therapy on patients with primary and secondary forms of AI as well as with respect to different formulas. This Mini-Review gives an overview of the current studies on GRT for primary and secondary AI and their impact on cognition. Strengths and weaknesses of the studies and their Implications for clinical daily routine are discussed with a special emphasis on practical considerations for the treating endocrinologist.

## 1 Introduction

Adrenal insufficiency (AI) describes a life-threatening condition caused by the inability of the adrenal glands to produce adequate amounts of cortisol. Adrenal glucocorticoid hormones are pivotal for maintenance of homeostasis and stress adaptation. AI can be divided into primary adrenal insufficiency (PAI), occurring at the level of the adrenals, secondary adrenal insufficiency (SAI), caused by dysfunction of the pituitary gland and tertiary adrenal insufficiency (TAI) which develops as a consequence of exogenous steroid treatment.

In Western countries PAI is a rare disease with a prevalence of 93 to 126 cases per million (1). It is mainly caused by an autoimmune disorder, called Addisons`s disease, which is responsible for > 80% of PAI cases. SAI is mainly caused by pituitary adenomas or other pituitary pathologies, including causes such as posttraumatic or radiation-induced pituitary dysfunction (2). The overall prevalence of SAI is about 455 cases per million (1, 3). TAI is the most common type of AI and usually caused by sudden withdrawal from long-term exogenous steroid use in patients, e.g., treated for inflammatory disorders. AI of any origin leads to a lack of endogenous glucocorticoids (GC) with typical clinical symptoms: unintentional weight loss, hypotension, fatigue, hyponatremia, muscle and abdominal pain (4). If untreated, symptoms can culminate to a potentially lethal event called adrenal crisis (5).

Patients with AI require life-long glucocorticoid replacement therapy (GRT). State of the art is treatment with hydrocortisone (15-25 mg HC), given in two or three doses daily with the highest dose in the morning (6). Although aiming to mimic the physiological pattern of cortisol release as closely as possible by splitting the total daily dose into 2-3 portions, such a regimen is still unphysiological and cannot prevent a temporary hyper- and hypocortisolism (7) contributing to morbidity and reduced life expectancy of the afflicted patients (8–11). Cardiovascular disease is the main cause of death in AI patients (12), with comorbidities largely accounting for the increase in mortality. GC replacement therapy itself (in particular HC doses > 20 mg/d) adversely affects some of the well-known risk factors for cardiovascular disease (CVD), for example, obesity, hypertension, diabetes, and hyper- lipoproteinemia (8, 13, 14). Temporary hypocortisolism may lead to subsequent increase of inflammatory markers being involved in atherosclerosis such as Interleukin 1 (IL-1), Interleukin 6 (IL-6), and tumor-necrosis factor (TNF) (15–18).

Moreover, health-related quality of life (HRQoL) is consistently reduced in patients with AI, despite adequate GRT (19, 20). Given the brain’s density of mineralocorticoid (MC) and glucocorticoid (GC) receptors and their profound role in neuropsychological functioning (21), it seems plausible that the inadequate cortisol homeostasis in AI contributes to reduced QoL and might also negatively influence cognition in the afflicted patients. An inverted-U-shaped pattern between circulating glucocorticoids and cognitive function has been repeatedly demonstrated (22, 23), implying that too high – as in Cushing’s syndrome (CS) – but also too low - as in underreplaced or unphysiologically replaced AI – cortisol levels negatively affect i.e. memory acquisition and retention (24–26). On a similar note, already the acute modulation of human cortisol levels by inhibition of cortisol production has been shown to impair memory, with the effect being completely reversed by subsequent administration of HC (27, 28).

In patients with active Cushing’s disease (CD) or CS, the literature gives evidence for impairments in all neuropsychological domains, most consistently concerning memory and visuo-spatial processing (for an overview see 29). Interestingly, short- and long-term remission of the disease are not generally associated with a normalization of cognitive function (for an overview see 29), perhaps not only owing to the effects of former glucocorticoid excess on the brain, but also to unphysiological replacement regimens in patients with cured hypercortisolism and consecutive SAI. Additional, preliminary evidence that hypocortisolism could impede neuropsychological functioning stems from patients recovered from aneurysmal subarachnoid hemorrhage (SAH) in whom low basal cortisol levels were the first and often only predictors for impairments in several QoL domains assessing psychological aspects of well-being and depression, whereas physical aspects of QoL were predicted mainly be neurological recovery from the SAH (30).

Modified-release hydrocortisone (MR-HC) preparations, introduced in recent years to avoid rapid rises in cortisol levels and to mimic the circadian rhythm of cortisol more physiologically (31) have already been shown to exhibit positive effects on metabolic profiles and cardiovascular parameters (32). It is conceivable that they may exhibit also favorable effects on cognitive function in patients with AI. Against the background of the increasing availability of more physiological HC replacement regimens, it is the aim of the present mini-review to present the reader with an overview on the published literature on cognitive dysfunction in patients with AI on GRT. Emphasis will be placed on practical considerations for the treating endocrinologist and the identification of potential research gaps and open questions in the field.

## 2 Methods

This Mini-Review aims to highlight previous studies on GRT and cognitive function. To this end, we conducted a systematic search through PubMed for all articles until December 31, 2022. We searched for any combination of the terms “glucocorticoid replacement therapy/glucocorticoids/hydrocortisone” or “adrenal insufficiency”/”Addison” with catchphrases relating to “cognition”, “cognitive”, “cognitive function”, “neurocognitive”, “neuropsychological”, “memory”, “executive function”, “impaired cognitive function”, “cognitive dysfunction”, “cognitive disorder”. All articles reporting original data and review articles on this topic in peer reviewed journals were screened for inclusion into the present review. We also searched the reference lists of articles identified by this search strategy to prevent overlooking relevant articles. Studies reporting data in patients with known neurological disorders or children were excluded.

## 3 Results

From our literature search, 11 studies were identified to be included in the present Mini-Review. Seven studies with a total of 235 patients and 248 healthy controls investigated potential neuropsychological impairment in patients with PAI (26, 33–38). In most of the studies an extensive neuropsychological test battery, administered in person, was used, whereas one study (35) relied on a self-report questionnaire on cognition and a further one on telephone-administered cognitive testing (37). In sum, the studies found none to only mild cognitive deficits in the investigated patients regarding subscales of memory (26, 33, 34, 37), attention (38) and executive functions (26).

Interestingly, the lack of gross objectifiable impairment was accompanied in some studies by a high incidence of self-experienced problems, i.e., in executive function, memory, and impaired QoL (26, 33, 35). In two of the studies, primarily designed to investigate the impact of sleep disturbances of PAI patients on neuropsychological functioning and cognition, sleep disturbances in PAI patients were uncovered by means of self-rating questionnaires. The studies’ authors postulated that the mild impairments in verbal learning and memory seen in the patients with PAI were due to their disrupted sleep patterns (34, 35), whereas another one interpreted impaired attention in PAI patients as a neuroglycopenic feature of Addison’s disease (38).

Two further studies included not only PAI but also SAI patients as well as healthy controls. The AI patients were investigated in in-group comparisons with respect to GRT dose (> 20 mg/die) and etiology (PAI vs. SAI) and against healthy matched controls. The two studies, investigating different patient groups, yield contradictory results in terms of PAI/SAI comparison and dosing regimen: one found PAI patients to perform better on tasks assessing intellectual abilities and executive function (39) whereas the other was not able to uncover differences between the two patient groups (40), using the same test battery.

A further difference concerned cognitive functioning between patients on low- versus high-dose HC replacement, with Blacha finding a negative effect of high-dose GRT on several cognitive domains while Krekeler did not (39, 40). While the number of patients investigated in the studies is not so low, the studies did not account for age or psychiatric morbidity as potential confounders of their results.

The last three studies in the field do not investigate AI patients in comparison to healthy controls but aimed to study whether different GRT regimens (41, 42) or disease duration (operationalized as duration of HC replacement, 43) impacted on cognition in patients with PAI and/or SAI. The largest of the three investigated SAI patients in a double-blind cross-over design after 10 weeks on physiological high-dose versus low-dose GRT with an extensive neuropsychological test battery (41). Additionally, serum cortisol levels were obtained in order to confirm differences between high- and low dose regimens. In this well-designed study, no differences in cognitive performance between the two dose regimens were found. The other two studies investigate a sample of 14 PAI/SAI patients, once under the condition of a nocturnal HC infusion (42) and once with respect to duration of HC replacement (43). In both no significant differences in cognition were found between the respective study conditions. A detailed table which lists the studies described here, can be found as supplemental material.

## 4 Discussion

Although an important topic, only a few trials about cognitive function in AI are available. Here, we review a total of 11 studies that reported cognitive functioning in patients with PAI and or SAI in comparison to healthy controls or under various HC replacement conditions. Cognitive impairment in AI patients was in general only mild or non-existent. If present, it concerned attention, executive functioning and memory domains and was attributed by some authors to AI-associated features such as sleep disturbances or impaired hypoglycemic control (34, 35, 38). On the other hand, in some studies, AI patients performed as well as their matched controls. In stark contrast to the overall mild objectifiable deficits, the largest of the studies reviewed here and two further studies found a high prevalence of self-experienced impairments concerning cognition and executive functioning (26, 33, 35).

Thus, the thin, current body of evidence does not reflect the pathophysiological hypotheses outlined in the introduction of this paper, which may be explained by the limitations of the studies in the field. First, some were published by a limited number of research groups. Two were based on one patient group investigated under different conditions (42, 43), from others it cannot be excluded that patients of former studies had been included repeatedly, thus conducting subsample analyses. Further conceptual weaknesses include a selection bias, especially in those studies investigating patients with PAI and SAI (39, 40) or the study by Van´t Westeinde et al. who included patients with a young median age and controls with exceptionally high neuropsychological test scores (33). Furthermore, the number of investigated patients was, in general, rather low, ranging from 10 – 67 patients, the latter constituting an exceptionally large sample. To confound matters further, the construct of “cognitive function” has been operationalized in the literature by different neuropsychological test batteries administered in person, at best (26, 33, 34, 36, 39–43), by telephone (37) or self-rating questionnaires (35). Moreover, virtually none of the studies controlled for age or internal medicine comorbidities, which might also have an influence on cognitive performance, and some did not control for psychiatric comorbidities such as anxiety or depression. The latter is important, though, as it is well researched that depression as well as anxiety may influence cognitive functioning to a considerable extent (44, 45). Last, but not least, the included patients had largely different disease durations and GRT regimens. This might be, however, a potentially minor concern, as the current study situation does not uncover a consistent influence of disease duration or different GRT dosing regimens, including MR-HC, on cognition. Similar concerns about the study situation have also been voiced by Ramos-Levi (46).

Taken in sum, the current body of evidence seems at first sight to be a clinical conundrum for the practicing endocrinologist. From the authors’ point of view, the most important take-home message is that no gross objectifiable neuropsychological impairment in patients with replaced AI has been proven so far and that different dosing regimens do not seem to impact significantly on cognition. This is an important point since from our experience patients tend to associate higher HC doses with a better efficacy, while the contrary holds true for metabolic, cardiovascular, bone parameters (47–50) and even QoL (47, 51, 52). Additionally, it is important to keep in mind that self-experienced cognitive impairment in patients with AI is hardly reflected in objective measurements. From a practical point of view, it seems advisable to counsel patients on this discrepancy and reassure them that their illness and its treatment is, in the great majority cases, not associated with cognitive dysfunction per se. Secondly, AI patients voicing severe neuropsychological complaints should be screened for depression and treated accordingly, as cognitive impairment may be caused or amplified by psychiatric comorbidity. In those patients, in whom clinically relevant depression is excluded or adequately treated and in whom cognitive disorders relevant to everyday life are present, neurological referral is advisable.

From a scientific point of view, large-scale studies, controlled for age, psychiatric and internal medicine comorbidities and GRT regimens will certainly expand our knowledge in the field. Additionally, the discrepancy between subjective and objectifiable cognitive impairment in AI patients deserves further research, especially against the background of designing therapeutic interventions. As large patient numbers are required to conduct discriminative statistics and interpret the results, the neuropsychological test batteries should include robust tests with no or little interrater variability paving the way for multicenter study designs that will allow to investigate enough patients with this rare disease.

## Author contributions

BH: concept/design, data analysis/interpretation, drafting article, and approval of article. PK: critical revision of article and approval of article. IK-A: concept/design, data analysis/interpretation, drafting article, and approval of article. All authors contributed to the article and approved the submitted version.
