# Supplementary material for: Glucocorticoid replacement therapy for primary and secondary adrenal insufficiency and their impact on cognition
Source: Front Endocrinol (Lausanne). 2023 Mar 17;14:1153595. doi: 10.3389/fendo.2023.1153595 (PMC10064134; doi:10.3389/fendo.2023.1153595)
Supplement: Supplementary file 1 [file Table_1.docx]

**Supplementary Table 1: Studies on cognitive functioning in patients with adrenal insufficiency**

| **Study** | **Design and patients** | **Measures** | **Main Findings** | **Comments** |
| --- | --- | --- | --- | --- |

**Studies on patients with PAI vs. healthy controls**

| Van´t Westeinde et al (2022) | Cross-sectional  67 pts. with PAI on GRT vs. 80 healthy matched controls | - **Cognitive Tests** for verbal and non-verbal intellectual ability, learning, memory and executive functioning (WAIS-IV Vocabulary; Digit Span; Span Board Test; WMS-III Coding, Stroop Task, WMS-III List Learning Test)  - **Self-experienced problems with executive function** (BDEFS-SF)  - **Anxiety/depression** (HADS)  - **Fatigue** (MFI) | - Worse intellectual ability (p=0.042) and visuo-spatial working memory (p= 0.020) in PAI pts.; however, performance within average range compared to population norms  -PAI pts. reported more problems with executive functions (p= 0.030) and on the subscales self-organization (p=0.005) and emotion regulation (p= 0.017) than controls  - Significant interaction with sex for the total BDEFS-SF scale (p = 0.049) and the self-organization subscale ( p = 0.011), with only female patients reporting more problems on the total scale  - Self-experienced problems with executive functions in both sexes were associated with increased mental fatigue and lower GC replacement doses | - Extensive neuropsychological test battery  - Executive Function, fatigue and depression assessed by self-rating  - No exclusion of patients treated for mood disorders (e.g.depression), however patients on antidepressant medication were statistically considered  - Selection bias possible (high neuropsychological test scores of controls, young median patient age)  - Heterogenous GRT (52 IR-HC, 15 MR-HC) |
| --- | --- | --- | --- | --- |
| Henry et al. (2017) | Cross-sectional, repeated measures, experimental design with a wake and a sleep condition  10 pts. with PAI on conventional GRT vs. 10 healthy matched controls | - **Cognitive tests** evaluating declarative memory and procedural memory  (Rey-Auditory- Verbal Learning Test; Finger Tapping Test)  - **Psychiatric disorders** (Mini International Neuropsychiatric Disorders Interview)  - **Depression** (BDI-II)  - **Intelligence** (Shipley-2 Intelligence Test)  - **Sleep** (Actigraph and Pittsburgh Sleep Diary) | - PAI pts. experienced disrupted, poor-quality sleep compared to controls  - PAI pts. did not benefit from a period of sleep in terms of memory consolidation  - Impaired verbal learning and memory (p=0.07) | - Authors used latent variable models to describe associations between sleep and (neuro)psychological impairments in PAI patients  - Memory deficits in PAI may be associated with disrupted sleep pattern that interfere with memory consolidation  - Participants with BDI-II scores > 29 were excluded |
| Tiemensma et al. (2016) | 31 pts. with PAI vs. 31 healthy matched controls | **Cognitive tests** evaluating memory and executive functioning (WMS; Rey-Verbal Learning Test; Rey Complex Figure Test; FAS; DST; Stroop task; TMT A/B; SART; GIT-2) | - Patients with PAI performed worse on auditory and visual memory tasks (all p <0.024) and executive functioning tasks (all p <0.012).  - Postponement of morning HC showed no effect  - PAI pts. reported more difficulties with attention, memory and executive functioning than controls | - Mild cognitive deficits in PAI patients on long-tern HC  - Patients were excluded in case of any neurological diagnosis  - Presence of depressive symptoms as a confounder was not evaluated |
| Henry et al. (2015) | Cross-sectional  60 pts. with PAI vs. 60 healthy matched controls | **Self-report questionnaire on cognition** (Cognitive Failures Questionnaire)  **Quality of life** (SF-36)  **Depression** (BDI-II)  **Sleep** (PSQI) | - PAI pts. reported poorer QoL, more depressive symptoms, more sleep disruptions, more memory impairment than controls (statistically significant in several domains of the respective questionnaires)  - No direct effect of PAI, but significant effect of sleep disturbances on neuropychological functioning. | - Cognition only assessed by means of self-rating  - Study design intended to assess effect of sleep pattern on neuropsychological functioning  - Controlled for depression as a confounder (Bonferroni correction) |
| Schultebraucks et al. (2015) | Cross-sectional  30 pts. with PAI compared to 30 healthy matched controls- | - **Cognitive tests** evaluating executive function, concentration, verbal memory, visual memory, working memory and autobiographical memory (AVLT; the Rey complex figure test; Digit Span; the Stroop task;, ZVT; AMT) - **Depression** (PHQ-9) - **Mood, fatigue** (MDBF) | - No differences in executive function, concentration, working memory, verbal memory, visuospatial memory and autobiographical memory between PAI pts. and controls but significantly worse performance in verbal learning (p=.007)  - Significantly more depressive symptoms reported by PAI pts. (p= 0.014) | - No clinically relevant cognitive impairment in PAI pts. compared to controls  -Significantly more pts. with mood disorders in PAI group (Bonferroni correction) |
| Henry et al. (2014) | Cross-sectional  27 PAI pts. vs. 27 healthy matched controls | **- Brief test of adult cognition, administered by telephone** to assess episodic memory, working memory, executive functioning, reasoning and speed of processing  - **BDI-II (depression)** | - No significant differences on attention, executive functioning, reasoning and speed of processing subtests between pts. and controls  - PAI pts. performed significantly worse only on episodic memory subtest  - Patients with a longer duration of illness performed more poorly across all domains (n.s.) | - Controlled for depression as a confounder (Bonferroni-correction) |
| Klement et al. (2010) | Cross-sectional  10 pts. with PAI with acutely discontinued HC replacement vs. 10 healthy matched controls | - **Cognitive Tests** evaluating attention and memory (Stroop task)  **- Mood** **assessments**  - **Symptom scores**  **- Biochemical parameters** assessed before and after a high calorie free choice buffet meal (comfort food) and a low calorie salad meal | - Neuroglycopenic symptoms higher in PAI patients than in controls with improvement by comfort food in contrast to salad (p<0.04)  - Reduced attention and impaired mood in PAI pts. compared to healthy participants. Pattern partially reversed after free-choice intake of comfort food | - Impaired attention in comparison to control subjects in the specific study design interpreted as neuroglycopenic feature of Addison’s disease |

**Studies on patients with PAI/SAI vs. healthy controls**

| Krekeler et al. (2021) | Cross-sectional  20 MR-HC pts. with AI compared with regard to etiology (PAI/SAI) and GRT dose (>20 mg/d);  18 of these MR-HC pts. compared to 18 matched conventionally treated patients | - **Cognitive Tests** evaluating intellectual abilities and mindset, short-term memory, executive functioning, attention and psychomotor response capability (CFT20-R; MWT-A; DST; Digit Span; TMT-A; TMT-B; LCT; test battery for attentional performance)  - **Quality of Life** (ADDIQoL, SF-36)  - **Depression** (BDI)  - **Sleep** (PSQI, ESS) | - Controls demonstrated significantly better psychomotor activity (p=0.037)  and intellectual abilities/mindset (p= 0.031) than MR-HC treated patients  - PAI pts. performed better on tasks assessing intellectual abilities (p= 0.038) executive functioning (p= 0.026) than SAI pts.  - No significant impact of MR-HC dosage on cognition  - Better subjective quality of sleep reported by AI pts. on high dose MR-HC (p= 0.028) than low dose MR-HC | - Extensive neuropsychological test battery, 8 a.m., standardized assessment  - Small patient sample for multiple comparisons  - Depression assessed but not investgated as a confounder for cognitive function  - Differences between PAI and SAI pts. with regard to cognitive function |
| --- | --- | --- | --- | --- |
| Blacha et al. (2021) | Cross-sectional,  40 pts. with AI (21 PAI/ 19 SAI), all but 2 pts. on conventional GRT vs. 20 healthy matched controls | **- Cognitive tests** evaluating memory, executive functioning, attention, psychomotricity and general intellectual ability  (CFT20-R; MWT-A; DST; Digit Span; WAIS; TMT-A; TMT-B; LCT; test battery for attentional performance) | - Significantly prolonged reaction time (p=0.002), and impaired response to visual stimuli (p=0.005) in AI pts. compared to controls  - Negative effect of high-dose GRT (> 25 mg/d) on several cognitive domains (attention, visual motoric skills, executive functioning). | - Extensive neuropsychological test battery, 8 a.m, standardized assessment  - Heterogenous HC regimens  - No differences between PAI and SAI with regard to cognitive function  - Not controlled for depression as a confounder |

**Studies under specific conditions**

| Harbeck et al. (2016) | 14 pts. with PAI/SAI compared with regard to duration of HC replacement (≤15 years) | - **Cognitive tests** evaluating memory and executive functioning (WAIS; MWT-B; DST; LCT; digit span; TMT-A/B;  battery for attentional performance)  - **Quality of life** (unstandardized questionnaire and structured interview)  - **Depression** (BDI) | - No differences in cognition and QoL between pts. on long-term vs. short-term GRT | - Small sample size  - Heterogenous HC regimens  - Wide range of serum cortisol levels  - Depression assessed but not investigated as a confounder for cognitive function |
| --- | --- | --- | --- | --- |
| Werumeus Buning et al. (2015) | Double-blind cross-over  47 pts. with SAI, investigated after 10 weeks on physiological high-dose (0.4-0.6 mg/kg body weight/d) and low-dose (0.2-0.3 mg/kg/body weight/d) | **Cognitive tests** evaluating memory, attention, executive functioning and social cognition (RBMT; the 15 Words Test, Digit Span; the Rey Complex Figure test; the 15 Figures Test; test battery for attentional performance; FAS, TMT A/B, and the Reading the Mind in the Eyes Test) | No differences in cognitive performance were found between the two dose regimens | - Untreated growth hormone (GH) deficiency in 10 patients  - Serum cortisol measured to confirm differences between high and low-dose GRT regimens  - Patients with current psychiatric disorders were excluded |
| Harbeck et al. (2009) | 14 pts. with PAI (5) or SAI (9) | - **Cognitive tests** evaluating memory, executive functioning, attention (WAIS; MWT-B; DST; LCT) after nocturnal i.v. hydrocortisone and 2-4 weeks later during oral GRT  **- Biochemical parameters**  - **Quality of life** (SF-36)  - **Depression** (BDI) | - Mimicking physiological cortisol rise over night by HC infusion was not associated with better cognition or better QoL in patients with PAI and SAI | - Pilot study with small sample size  - Depression assessed but not investgated as a confounder for cognitive function |

**Abbreviations:**

ADDIQoL Addison’s-disease-specific-Quality-of- life-questionnaire

AMT Autobiographical Memory test

AVLT Neuropsychological testing comprised the auditory verbal learning test

BDEFS-SF Barkley Deficits in Executive Functioning Scale short form

BDI Beck-Depressions-Inventar

CFT20-R Culture- fair-test

DST Digit symbol test

ESS Eppworth-Sleeping- Scale

FAS Verbal Fluency Test

GIT-2 Groninger Intelligence Test 2

GRT Glucocorticoid replacement therapy

HADS The Hospital Anxiety and Depression Scale

HC Hydrocortisone

IR-HC Immediate- release hydrocortisone

LCT d2 letter cancellation test

MDBF Mehrdimensionaler Befindlickeitsfragebogen

MFI The multidimensional fatigue inventory

MR-HC Modified -release hydrocortisone

MWT A/B Mehrfachwahl Wortschatz Intelligenztest A/B

PAI primary adrenal insufficiency

PHQ-9 Patient Health Questionnaire

PSQI Pittsburgh-Sleep-Quality-Index

RBMT The Rivermead Behavioral Memory Test

SAI Secondary adrenal insufficiency

SART Sustained Attention to Response Task

SF-36 Short- Form-36

TMT-A/B Trail-Making-Test A and B

WAIS Wechsler Adult Intelligence Scale (WAIS)-IV

WMS Wechsler Memory Scale

ZVT Number-Combination test
